# Prevalence of negative emotional eating and its associated psychosocial factors among urban Chinese undergraduates in Hong Kong: a cross-sectional study

**DOI:** 10.1186/s12889-021-10531-3

**Published:** 2021-03-24

**Authors:** Katherine Y. P. Sze, Eric K. P. Lee, Rufina H. W. Chan, Jean H. Kim

**Affiliations:** Jockey Club School of Public Health and Primary Care, The Chinese University of Hong Kong, Prince of Wales Hospital, Shatin, New Territories Hong Kong SAR

**Keywords:** Eating behaviour, Emotional eating, University, Diet, Obesity

## Abstract

**Background:**

Emotional eating (EE), defined as eating in response to a range of emotions, has been previously associated with poor diet and obesity. Since there are limited data from non-Western populations, this study aims to examine the prevalence and factors associated with EE among urban Chinese university students.

**Methods:**

A cross-sectional study was conducted on 424 university students (aged 18–24 years) from two large universities in Hong Kong in 2019. Respondents completed an anonymous online questionnaire that contained background questions, an emotional eating subscale of the Dutch Eating Behaviour Questionnaire (DEBQ), and Depression Anxiety and Stress Scales (DASS-21). Two-sample independent t-test and multiple regression analyses were conducted to test the association of study variables with negative emotional eating.

**Results:**

There was over a three-fold higher likelihood of negative EE among females (14.8%) when compared with their male counterparts (4.5%) (OR = 3.7, *p* < 0.05). Having at least mild depressive symptoms was the only independent factor associated with negative EE among males (OR = 10.1) while for females, negative EE was independently associated with not having a romantic partner (OR = 3.45), having depressive symptoms (OR = 44.5), and having at least mild stress (OR = 5.65). Anxiety levels were not independently associated with negative EE for either gender. Both male and female students with negative EE had significantly lower self-perceived health scores, higher body mass index, and lower life satisfaction scores.

**Conclusions:**

This study revealed that negative EE is prevalent among female Chinese university students and not uncommon among male students. Management of negative EE should be included as a component of university mental health promotion programmes in the region.

## Background

Across cultures, university is often a highly stressful developmental period for young adults due to various academic and social pressures. In the literature, there exists an extensive body of research examining maladaptive behaviours in college students such as substance abuse and excessive alcohol consumption [1–4]. Although comparatively less studied, emotional eating (EE), eating in response to a range of emotions [5], has been gaining interest among college health researchers in recent years [5–10]. Both positive and negative emotions can lead to greater food intake and overeating [11–14]. Yet studies suggest that positive EE and negative EE are different constructs that are driven by different mechanisms. Positive EE is associated with external influences, higher intensity of positive emotions, lower likelihood of emotional regulation difficulties; moreover positive EE does not necessarily reflect disordered eating [13–17]. Positive emotions such as happiness, particularly in the context of celebrations and socializing, can trigger hedonic overeating and was noted to enhance the pleasure of food [18, 19]. On the other hand, negative EE is associated with emotional regulation difficulties, internal triggers such as poor mood, longer duration of negative feelings and higher risk of eating pathology such as binge eating [13–17]. According to the theory of maladaptive behaviour and affect regulation, individuals with negative EE consume food for the hedonic experience in an attempt to relieve negative emotions; however, excessive food consumption can, in turn, lead to negative consequences and effects (e.g. obesity and guilt), providing cues for further food consumption and lead to a vicious cycle [20, 21]. In fact, similar to many recreational drugs, food consumption can stimulate dopamine secretion in the brain, which controls the reward-related behaviours [22]. While episodes of EE may occur in response to positive emotions, studies of health-related effects of EE have predominantly examined EE behaviour as a response to negative emotional states such as stress, depression, boredom, and anxiety [6–10, 22, 23]. Studies from Western countries have reported that negative EE prevalence among college students ranges from 8.9 to 56% and negative EE was noted to be more common in females and those with higher levels of stress, depression, and anxiety [22, 24–28]. Since individuals with negative EE tend to binge eat and consume highly caloric foods, negative EE has been associated with weight gain, overweight/obesity, difficulty in losing weight and sustaining weight loss, and even metabolic disorders across many age groups [28]. Negative EE may be on the intermediate pathway between dysphoric mood and excessive food consumption, hence, management of negative EE may be a possible target for the prevention of obesity [29].

There have been very few studies examining EE in the Chinese populations although its cultural meanings of food and eating habits differ notably from the Western populations. Unlike many Western cultures, food consumption is a highly communal behaviour in Chinese society and eating alone is not a common practice [30]. Moreover, snacking between meals is not commonly seen in Chinese eating culture [30]. In the past two decades, however, there have been major shifts towards increased snacking and greater consumption of high-calorie food in economically developed areas of China, particularly among individuals with a higher socioeconomic status [31, 32]. Previous research conducted in Chinese adolescents living at home in Eastern China noted a negative EE prevalence of 18.6% among males and 36% among females [33]. In cities such as Hong Kong and other urban areas of China, university students may thereby represent a potential high-risk group for negative EE. In these regions, university is often the first time when young adults may live away from their parents and consume the majority of their food intake outside the family home whereas those entering the work force after secondary school would typically continue to live at home [34, 35].

There are few studies about negative EE in Chinese university populations. Recently conducted studies from central China have reported negative EE prevalence ranging from 14.5 to 52.7% among undergraduate students [36, 37]. These findings suggest that negative EE may be prevalent among Chinese youths but the generalizability to other urban areas is unknown. University life has been noted to be extremely stressful in Hong Kong [24, 38, 39]. In comparison to other cities in China, Hong Kong, a former British colony, has a long history of Westernized fast-food and convenience store pre-packaged foods that are highly conducive to impulsive food consumption. It is unclear whether the prevalence of negative EE is higher in cities such as Hong Kong and it is not known which emotions trigger episodes of negative EE. Moreover, there is limited information about its associations with psychosocial factors such as life satisfaction and self-perceived health and very limited data on the associations with physical health indicators such as body weight in young Chinese adults.

In order to inform health interventions for university students in the region, this cross-sectional study aimed to determine the prevalence of negative EE among university students in Hong Kong and to see whether the prevalence of negative EE differs by gender. In addition to determining the sociodemographic predictors of negative EE, this study will examine whether self-reported symptoms of depression, anxiety, and stress are significantly associated with negative EE in male and female students. We will also examine whether negative EE is associated with various self-reported indicators of physical health and mental well-being to provide insight into possible outcomes of negative EE. In addition to informing regional university health promotion, these findings can also provide a springboard for more focused, theory-based studies in the future.

## Methods

### Participants

This study recruited university students through face-to-face intercept survey in large public areas of the two major universities in Hong Kong (The University of Hong Kong and The Chinese University of Hong Kong) between April and June 2019. The researchers also sent out a mass email with a link to the online survey in August 2019. The normative study period in Hong Kong university is typically 4–6 years, depending on the study major, and the normative age of university enrolment is 18 years of age. In order to capture undergraduate students, participants were only included if they were (i) of 18–24 years of age and (ii) studying an undergraduate degree. International and exchange students were excluded from this study. Sample size calculations were conducted based on an estimated 20% EE prevalence (α = 0.05). In order to obtain a precision of 4%, a sample size of 385 was required. The study recruited 424 participants to allow for possible missing data and non-completion of the survey. All participants were invited to fill in a standardized questionnaire on the Google survey platform (details see below). Ethical approval was obtained from the Survey and Behavioural Research Ethics Committee of the university sponsoring the study prior to participant recruitment (Approval #043–19).

### Instruments

#### Dutch eating behaviour questionnaire (DEBQ)

The 13-item Emotional Eating Subscale of the Dutch Eating Behaviour Questionnaire (DEBQ) was the primary outcome of the current study and was one of the most commonly used instruments to examine negative EE in research [40]. Each statement in the DEBQ was rated with a 5-point Likert scale (ranging from 1 “never” to 5 “very often”). The scores from each statement were summated and averaged to obtain the final score. From a study of Chinese adolescents, the DEBQ was previously shown to have high internal consistency (Cronbach’s alpha = 0.964) [41]. Higher DEBQ scores suggest more severe symptoms of negative EE and scores > 3.25 are recommended to be used in research to identify individuals with negative EE [40].

#### Depression, anxiety, and stress scales (DASS-21)

Depression Anxiety and Stress Scales (DASS-21) consist of depression, anxiety, and stress subscales that each have seven items. All three subscales of DASS-21 had high internal consistency; the Cronbach’s alphas were 0.83, 0.80, 0.82, and 0.92 for depression, anxiety, stress, and overall scale respectively in Chinese students [42]. Each statement was rated on a 4-point Likert scale; the score of the subscales can be calculated by summation of scores from respective individual items, with higher scores indicative of higher levels of depression, anxiety, and stress. Participants with depression score > 9, anxiety score > 7, and stress score > 14 are identified as having depression, anxiety, and stress symptoms, respectively [42]. The distribution of these scores in our sample is shown in Table 1.

**Table 1:** Background characteristics of the study sample by gender (*n* = 424)

	Males (***n*** = 201) % (n)	Females (***n*** = 223) % (n)	***P***-value*	All (***n*** = 424) % (n)
**Mean Age**	20.4 (SD = 1.6)	20.0 (SD = 1.6)	0.014	20.2 (SD = 1.6)
**Place of birth**			0.593	
***Hong Kong***	84.1% (169)	80.3% (179)		82.1% (348)
***China***	10.4% (21)	13.0% (29)		11.8% (50)
***Other***	5.5% (11)	6.7% (15)		6.1% (26)
**Parent’s education level**			0.911	
***Up to high school***	58.21% (117)	58.74% (131)		58.49% (248)
***University or more***	41.79% (84)	41.26% (92)		41.51% (176)
**Faculty**			< 0.001	
***Arts/ Education***	5.47% (11)	30.9% (69)		18.9% (80)
***Business***	18.4% (37)	13.5% (30)		15.8% (67)
***Social Science***	8.46% (17)	12.6% (28)		10.6% (45)
***Science/Engineering***	40.3% (81)	14.8% (33)		26.9% (114)
***Law***	6.0% (12)	4.04% (9)		5.0% (21)
***Medicine/Dentistry***	21.4% (43)	24.2% (54)		22.9% (97)
**Religious affiliation**			0.533	
***Protestant***	18.9% (38)	23.3% (52)		21.2% (90)
***Catholic***	4.0% (8)	2.7% (6)		3.3% (14)
***Buddhist***	3.0% (6)	1.8% (4)		2.4% (10)
***No religion***	74.1% (149)	72.2% (161)		73.1% (310)
**Housing**			0.548	
***Live at home***	43.3% (87)	46.2% (103)		44.8% (190)
***Dorm or elsewhere***	56.7% (114)	53.8% (120)		55.2% (234)
**Part-time job**			0.522	
***Yes***	50.3% (101)	53.4% (119)		51.9% (220)
***No***	49.8% (100)	46.6% (104)		48.1% (204)
**Boyfriend/girlfriend**			0.680	
**Yes**	57.2% (115)	59.2% (132)		58.3% (247)
***No***	42.8% (86)	40.8% (91)		41.8% (177)
**Exercising at least moderately**			0.049	
***Yes***	34.3% (69)	25.6% (57)		29.7% (126)
***No***	65.7% (132)	74.4% (166)		70.3% (298)
**Mean Life satisfaction score (SD)**^**a**^	3.6 (SD = 0.6)	3.6 (SD = 0.6)	0.959	3.6 (SD = 0.6)
**Mean Perceived health score** ^**b**^**(SD)**	3.9 (SD = 0.7)	3.6 (SD = 0.7)	< 0.001	3.7 (SD = 0.7)
**Mean Body Mass Index (SD)**	22.0 (SD = 2.4)	20.6 (SD = 2.8)	< 0.001	21.3 (SD = 2.7)
**Mean Emotional Eating Score (SD)**	1.87 (SD = 0.7)	2.42 (SD = 0.9)	< 0.001	2.16 (SD = 0.8)
***DEBQ score*** **≤** ***3.25***	95.5% (192)	85.2% (190)	< 0.001	90.1% (382)
***DEBQ score > 3.25***	4.5% (9)	14.8% (33)		9.9% (42)
**DASS-21 Depression levels**			< 0.001	
***Normal (0–9 points)***	84.1% (169)	65.9% (147)		74.5% (316)
***Mild (10–13 points)***	10.5% (21)	13.0% (29)		11.8% (50)
***Moderate (14–20 points)***	4.0% (8)	15.3% (34)		9.9% (42)
***Severe (21–27 points)***	1.49% (3)	4.93% (11)		3.3% (14)
***Extremely Severe (28+ points)***	0.0% (0)	0.90% (2)		0.47% (2)
**DASS-21 Anxiety levels**			0.002	
***Normal (0–7 points)***	88.6% (178)	72.6% (162)		80.2% (340)
***Mild (8–9 points)***	2.5% (5)	6.7% (15)		4.7% (20)
***Moderate (10–14 points)***	6.5% (13)	13.0% (29)		9.9% (42)
***Severe (15–19 points)***	0.5% (1)	2.2% (5)		1.4% (6)
***Extremely Severe (20+ points)***	2.0% (4)	5.4% (12)		3.8% (16)
**DASS-21 Stress levels**			< 0.001	
***Normal (0–14 points)***	94.5% (190)	84.8% (189)		89.4% (379)
***Mild (15–18 points)***	0.50% (1)	7.2% (16)		4.0% (17)
***Moderate (19–25 points)***	2.0% (4)	7.6% (17)		5.0% (21)
***Severe (26–33 points)***	3.0% (6)	0.4% (1)		1.7% (7)
***Extremely Severe (34+ points)***	0.0% (0)	0.0% (0)		0.0% (0)

*Chi-square *p*-value for categorical variables and t-test *p*-values for continuous variables

^a^Life satisfaction score possible range from 1 to 5

^b^Perceived health score possible range from 1 to 5

### Covariates and secondary outcomes

Demographic and background data including age (years), sex (male/female), place of birth (Hong Kong, China, other countries), faculty of study, and presence of romantic relationship (yes/no) were collected. The instrument also asked whether the student engaged in weekly exercise (yes/no) which has been shown to have adequate validity and reliability [43]. As the questionnaire was conducted in an open area in the universities without measurement instruments, the weight and height of the participants were self-reported. Body mass index (BMI) was calculated by weight in kilograms divided by height in meters squared and obesity was defined as body mass index of > 23 kg/m^2^, which was the international cut-off for Southeast Asians [44].

In order to maintain the brevity of the survey instrument, life satisfaction, and self-perceived health were each asked using a single question: “You are satisfied with your life” and “You perceive yourself as healthy”, respectively. These items used a 5-point Likert Scale response (ranging from “1”: totally disagree to “5”: totally agree) to the statements. Both items have been used extensively in health research and shown to have adequate psychometric qualities [45, 46].

### Statistical analysis

Demographic data and psychosocial factors among female and male students were described by the mean and standard deviation (SD) and by percentage and absolute number for continuous and categorical variables respectively. We stratified all data analyses by gender since past studies have noted gender differences in the prevalence and predictors of negative EE [25, 47]. The prevalence estimates of negative EE were shown for males and females separately with their respective 95% confidence intervals (CI). For all univariable analyses, the relationship between negative EE and various psychosocial and health factors was explored by Chi-Square and Fisher-exact test as appropriate.

In order to determine variables independently associated with negative EE, all variables that demonstrated an association with *p* < 0.20 in the unadjusted analysis were considered as candidate variables in the backward multiple logistic regression model. We used a conservative cut-off of *p* < 0.20 since the traditional cut-off of *p* < 0.05 will often miss independent factors [48]. Mann-Whitney U test was performed to compare the mean BMI, self-perceived health score, and life satisfaction scores while χ^2^-test was used to compare the prevalence of obesity in students with and without negative EE. Statistical significance was set at α. = 0.05 and all data were analysed using SPSS v25.0 (IBM Corp. Released 2017. IBM SPSS Statistics for Windows, Version 25.0. Armonk, NY: IBM Corp).

## Results

### Participants characteristics

Of the 424 participants, a similar number of males (*n* = 201) and females (*n* = 223) were recruited. The mean age for the study sample was 20.2 years (SD = 1.6). Of the sample, the majority were born in Hong Kong (82.1%), had no religion (73.1%), were living away from home (55.2%), had a part-time job (51.9%), had a boyfriend/girlfriend (58.3%), and had no/little exercise (70.3%) (Table 1). The vast majority of students had DASS-21 subscale scores indicating normal levels of depressive symptoms (74.5%), anxiety symptoms (80.2%), and stress symptoms (89.4%). Male students reported better perceived health, higher mean BMI, and lower emotional eating score (*p* < 0.001), while female students reported higher depression, anxiety, and stress scores (*p* < 0.05) (see Table 1).

### Prevalence of emotional eating

In our university student sample, 9.9% (95% CI: 7.1–12.8%) of university students were classified as an individual with negative EE. Female students revealed a much higher prevalence of negative EE (14.8%; 95% CI: 10.1–19.5%) than male students (4.5%; 95% CI: 1.6–7.4%). Females had more than three times the likelihood of having negative EE when compared with males (OR = 3.7, *p* < 0.05).

### Psychosocial factors associated with emotional eating

In the univariable analysis, there was a higher likelihood of negative EE in male students who lived at home (odds ratio (OR = 5.0), had a part-time job (OR = 3.73), had depressive symptoms (OR = 23.4), had anxiety symptoms (OR = 12.1), and had stress symptoms (OR = 21.1). In the backward multiple regression analysis of these candidate variables, EE was significantly associated only with the presence of depressive symptoms in male students (OR = 10.1).

In female students, there was a statistically significantly higher likelihood of negative EE among women who were not in a romantic relationship (OR = 3.51), had depression symptoms (OR = 106), had anxiety symptoms (OR = 9.2), and had stress symptoms (OR = 19.3) in the univariable analysis. The backward multiple regression of these variables revealed that negative EE was independently associated with the absence of a romantic partner (OR = 3.45), having at least mild depressive symptoms (OR = 44.5) and having at least mild stress (OR = 5.65) in female students (Table 2).

**Table 2:** Factors associated with negative emotional eating in Chinese university students (*n* = 424)

Factors	Male students (***n*** = 201)			Female students (***n*** = 223)		
	Emotional Eating % (n)	Unadjusted OR (95% CI)	Multivariable OR (95% CI)	Emotional Eating % (n)	Unadjusted OR (95% CI)	Multivariable OR (95% CI)
**Age**						
*< 21 years of age*	1.9% (2)	1.00	1.00	15.7% (22)	1.00	–
*≥ 21 years of age*	7.4% (7)	4.14 (0.84–20.4)^†^	2.71 (0.47–15.7)	13.3% (11)	0.82 (0.38–1.79)	
**Place of birth**						
*Hong Kong*	4.1% (7)	1.00	–	14.5% (26)	1.00	–
*Foreign-born*	6.3% (2)	1.54 (0.31–7.79)		15.9% (7)	1.11 (0.45–2.76)	
**Parental education**						
*Up to high school*	4.3% (5)	1.00	–	17.6% (23)	1.00	–
*University or more*	4.8% (4)	1.12 (0.29–4.30)		10.9% (10)	0.57 (0.26–1.27)	
**Area of study**						
*Non-Science majors*	2.6% (2)	1.00	–	11.8% (16)	1.00	–
*Science majors*	5.6% (7)	2.24 (0.45–11.1)		19.5% (17)	1.82 (0.87–3.83)	
**Religiosity**						
*No religion*	4.7% (7)	1.00	–	15.5% (25)	1.00	–
*Has religion*	3.8% (2)	0.81 (0.16–4.04)		12.9% (8)	0.81 (0.34–1.90)	
**Housing**						
*Live at home*	8.0% (7)	1.00	1.00	14.6% (15)	1.00	–
*Dorm/Elsewhere*	1.8% (2)	0.20 (0.04–1.01)^†^	0.33 (0.06–1.94)	15.0% (18)	1.04 (0.49–2.18)	
**Part-time job**						
*Yes*	2.0% (2)	1.00	1.00	17.6% (21)	1.00	–
*No*	7.0% (9)	3.73 (0.76–18.4)^†^	2.01 (0.33–12.3)	11.5% (12)	0.61 (0.28–1.31)	
**In a romantic relationship?**						
*Yes*	4.3% (5)	1.00	–	8.3% (11)	1.00	1.00
*No*	4.7% (4)	1.07 (0.28–4.12)		24.2% (22)	3.51 (1.61–7.67)^‡^	3.45 (1.18–10.0)^*^
**DASS-Depression**						
*Normal score*	1.2% (2)	1.00	1.00	0.7% (1)	1.00	1.00
*Depressive symptoms*	21.9% (7)	23.4 (4.60–119)^§^	10.1 (1.51–67.4)^*^	42.1% (32)	106 (14.1–799)^§^	44.5 (5.58–356)^§^
**DASS-Anxiety**						
*Normal score*	2.2% (4)	1.00	1.00	6.2% (10)	1.00	1.00
*Anxiety symptoms*	21.7% (5)	12.1 (2.98–49.1)^§^	1.13 (0.13–9.75)	37.7% (23)	9.20 (4.04–21.0)^*^	0.86 (0.28–2.68)
**DASS-Stress**						
*Normal score*	2.6% (5)	1.00	1.00	6.9% (13)	1.00	1.00
*Stress symptoms*	36.4% (4)	21.1 (4.64–96.3)^§^	3.43 (0.37–32.3)	58.8% (20)	19.3 (7.98–46.9)^§^	5.65 (1.93–16.6)^‡^

*P < 0.05; †*P* < 0.10; ‡*P* < 0.01; §*P* < 0.001; −-: the variable did not meet the *p* < 0.20 threshold for inclusion as a candidate variable for the backwards multiple regression model. For the multiple regression model, Odds Ratio (95% CI) are shown for non-significant candidate variables prior to being dropped from the final multiple regression model

### Associations of emotional eating with bodyweight, perceived health, perceived life satisfaction and obesity

Both male and female university students with negative EE reported higher BMI, poorer self-perceived health, and lower life satisfaction than their non-EE counterparts (*p* < 0.05) (Tables 3 and 4). Although obesity was more prevalent in students with negative EE for both genders, females showed a statistically significant difference (48.5% versus 11.1% in non-EE females; *p* < 0.001) while males students showed a marginally significant difference (66.7% versus 30.7% in non-EE males; *p* = 0.061) (non-tabulated).

**Table 3:** Body mass index, health perceptions and life satisfaction scores by negative emotional eating status for males (*n* = 201)

			Mean (SD)	***P***-value
**Males**	Individuals with negative EE (*n* = 9)	*Body Mass Index*	24.0 (2.5)	0.024
	Individuals without negative EE (*n* = 192)		21.9 (2.4)	
	Individuals with negative EE (*n* = 9)	*Perceived health score*	3.00 (0.9)	0.001
	Individuals without negative EE (*n* = 192)		3.89 (0.6)	
	Individuals with negative EE (*n* = 9)	*Life satisfaction score*	3.00 (0.9)	0.013
	Individuals without negative EE (*n* = 192)		3.65 (0.6)	

Individuals with negative EE (Mean DEBQ score > 3.25) versus Normal negative EE score (Mean DEBQ score ≤ 3.25); higher self-perceived health scores and life satisfaction scores indicate higher levels of health and life satisfaction; *p*-value assessed with Mann-Whitney U Test

**Table 4:** Body mass index, health perceptions and life satisfaction scores by emotional eating status for females (*n* = 223)

	DEBQ Score		Mean (SD)	***P***-value
**Females**	Individuals with negative EE (*n* = 33)	*Body Mass Index*	22.3 (4.0)	0.01
	Individuals without negative EE (*n* = 190)		20.3 (2.4)	
	Individuals with negative EE (*n* = 33)	*Perceived health score*	2.94 (0.6)	< 0.001
	Individuals without negative EE (*n* = 190)		3.69 (0.6)	
	Individuals with negative EE (*n* = 33)	*Life satisfaction score*	3.09 (0.6)	< 0.001
	Individuals without negative EE (*n* = 190)		3.71 (0.6)	

Individuals with negative EE (Mean DEBQ score > 3.25) versus Normal negative EE score (Mean DEBQ score ≤ 3.25); higher self-perceived health scores and life satisfaction scores indicate higher levels of health and life satisfaction; *p*-value assessed with Mann-Whitney U Test

## Discussion

This study revealed that nearly one in ten Chinese students in this Hong Kong university sample were classified as engaging in negative EE. Although students were sampled from only two universities, their demographic and lifestyle characteristics are not expected to differ from other tertiary institutes. This study, therefore, provides a rough estimate of the prevalence of negative EE among Hong Kong university students. Direct comparison with other studies, however, must be made with caution. Due to differences in the instruments used to assess negative EE, the underlying constructs being measured may not be directly comparable. While in our study, the DEBQ instrument assesses the motivation to eat in response to negative emotions like stress, a recently published study from central China that examined negative EE as a form of internal disinhibition or loss of control reported a prevalence of 52.7% [37]. By contrast, another recent study from China noted that 14.5% of its undergraduate sample reported emotional overeating in response to a smaller number of negative emotions rather than the range assessed by the DEBQ [36]. Use of other scales such as the Emotional Eating Scale (EES) which includes a wider range of 25 possible emotional triggers for negative EE would likely have increased the prevalence estimates of this study [23].

Our results confirmed findings from international studies that females were much more likely to have negative EE as compared with their same-age male counterparts [25–28, 36, 37]. Studies from China have also shown negative EE to be more prevalent among females. Although the reasons for the strong gender differential are unclear from this analysis, past studies have noted that females are less likely to eat in response to hunger/satiety cues than males but were more responsive to external signals (e.g. stress) or rules (dieting) [49]. A previous study also noted that females have genetic predisposition for higher impulsivity and higher reward sensitivity, which are associated with dopamine dysregulation during comfort eating [50]. Our study was one of the first Chinese studies to note that negative EE behaviours and its relationship with psychosocial factors differ between genders. In male students, negative EE was only associated with greater depression levels, while negative EE was also associated with higher stress levels among females. Having a romantic partner was protective for negative EE among female students only. Due to the cross-sectional nature of this study, it is unclear whether close interpersonal interactions from a romantic relationship may reduce reliance on emotional eating as a coping mechanism or if those who partake in behaviours such as negative EE are less likely to form these relationships. However, our study did not show that anxiety was independently associated with negative EE. It may reflect that negative EE is not a common coping/affect regulation mechanism for anxiety in Chinese university students or that the sample size was insufficient to detect an association. Additionally, unlike many other EE instruments, the EE subscale of the DEBQ instrument does not allow for the examination of overeating in relation to specific negative mood states such as anxiety or anger. The EE subscale of the DEBQ instead broadly defines negative EE in response to a wide range of negative emotions and the single subscale score may therefore not be correlated strongly with a specific mood state such as anxiety. Individual subscales of EE instruments that specify types of EE (such as emotional eating for anger or boredom) have been noted to be correlated with measures of poorer psychological well-being [51, 52]. Use of another EE instrument such as Emotional Eating Scale (EES) which is comprised of three subscales (anxiety, depression, anger/frustration) by contrast, may have resulted in significant associations with the DASS-21 anxiety score in our study [23].

Our findings reveal that negative EE is associated with poorer mental health, higher BMI, lower life satisfaction, and lower perceived health, which in turn, have been linked with a multitude of other health conditions. The results thereby suggest that screening and management of EE are needed among Chinese university students. Clinicians may consider screening for negative EE, especially when seeing young adults with negative emotions, and offer appropriate counselling and interventions. However, despite being associated with adverse physical and psychological consequences, there is a lack of guideline-based treatments for EE in Hong Kong and worldwide. As negative EE was conceptualized as a poor stress-coping strategy, treatments that enhance emotional coping skills may reduce negative EE [53]. For instance, the latest meta-analysis suggested that mindfulness-based interventions could reduce EE and body weight [54]. However, there is a lack of similar studies in the non-Western populations. High-quality randomized controlled trials will be needed to examine treatment modalities in the Chinese population.

The strength of the current study includes the use of widely validated questionnaires (DEBQ and DASS-21) and a large array of psychosocial and lifestyle factors. To the authors’ knowledge, the relationship between EE and self-reported health was not previously examined. Although the data were collected through self-report and may be prone to social desirability bias, the use of an anonymous questionnaire is likely to have limited social desirability in answering sensitive questions. Although the sample size was modest, this study has a larger sample size than most of the existing studies conducted on university populations [5–7, 9, 10, 25, 26, 55].

This study has several limitations. First, due to the cross-sectional study design, the direction of many of the significant associations (e.g. negative EE and depression) cannot be ascertained conclusively. Second, even though the sociodemographic background and general lifestyle habits of the study sample are not expected to differ appreciably from other undergraduates in the region, students from these two universities may not be fully representative of all tertiary institute students in Hong Kong. Third, although our sample size was adequate for negative EE prevalence estimations, only nine male participants engaged in negative EE and this could limit the power to detect the association between EE and psychosocial factors among male students. Moreover, spurious findings cannot be ruled out in a multivariable regression model that includes multiple covariates. Fourth, single item measures on life satisfaction, perceived health, and physical activity were used for capturing secondary outcomes in our study but future studies should consider using multi-item scales for detailed assessment. Fifth, in examining factors associated with EE, there were potential confounding variables that were not covered in this study such as social support and the presence of eating disorder psychopathology.

Future research in non-Western populations should address some of the ongoing methodological issues in EE research. Although self-reported scales are commonly used to assess EE, the validity of the DEBQ has been debated in some past studies [56, 57]. Specifically, there has been conflicting evidence of increased food intake in individuals scoring high on self-reported emotional eating scales such as the DEBQ [56–58]. Researchers have also questioned whether such scales simply measure uncontrolled eating rather than emotional eating [56]. Future studies in Chinese populations could thereby explore alternative methods of assessing negative EE such as ecological momentary assessment and real-time sampling of participant’s thoughts and behaviours, in conjunction with concurrent dietary assessments. As there is comparatively little research on disordered eating patterns in East Asian populations, future studies should thereby examine whether individuals with negative EE also engage in disordered eating behaviours. Finally, as most studies have only examined negative EE in relation to overeating, future studies in Chinese population should also include EE in response to positive emotions as well as undereating behaviours for a better understanding of this phenomenon.

## Conclusion

Negative emotional eating is an underappreciated risk behaviour in urban Chinese university students. Given its associations with negative emotions and other aspects of health, screening and management of EE may improve multiple areas of health and well-being. More studies in young Chinese adult populations are thereby needed to determine the best treatment strategies for negative EE.

## Data Availability

The datasets used and/or analysed during the current study are available from the corresponding author on reasonable request.

## Abbreviations

BMI: Body mass index
CI: Confidence interval
EE: Emotional eating
DEBQ: Dutch Eating Behaviour Questionnaire
DASS-21: Depression Anxiety and Stress Scale
SD: Standard deviation
